# SCARF1: a multifaceted, yet largely understudied, scavenger receptor

**DOI:** 10.1007/s00011-018-1154-7

**Published:** 2018-05-03

**Authors:** Daniel A. Patten

**Affiliations:** National Institute for Health Research Birmingham Liver Biomedical Research Unit and Centre for Liver Research, 5th Floor Institute of Biomedical Research, Institute of Immunology and Immunotherapy, University of Birmingham, Birmingham, B15 2TT UK

**Keywords:** SCARF1, SREC-I, Innate immunity, Leukocyte adhesion molecule

## Abstract

**Background:**

As is a prerequisite of belonging to the scavenger receptor super family, SCARF1 (scavenger receptor class F, member 1) is known to play a key role in the binding and endocytosis of a wide range of endogenous and exogenous ligands.

**Findings:**

Unlike most scavenger receptors, SCARF1 is an essential protein, as SCARF1-deficient mice exhibit a severe resting phenotype in which they develop systemic lupus erythematosus (SLE)-like disease, thus highlighting the importance of SCARF1-mediated clearance of apoptotic host cells in homeostasis. In addition, a number of other roles in homeostasis and disease pathology have also been suggested, including roles in both innate and adaptive immunity; however, the majority of these studies have utilised transfected cell lines engineered to ectopically express SCARF1 and very few have utilised in vivo or ex vivo approaches.

**Conclusion:**

This review summarises our current knowledge on SCARF1 biology and reflects on future directions for research on this multifaceted, yet largely understudied, scavenger receptor.

## Introduction

**Fig. 1:**
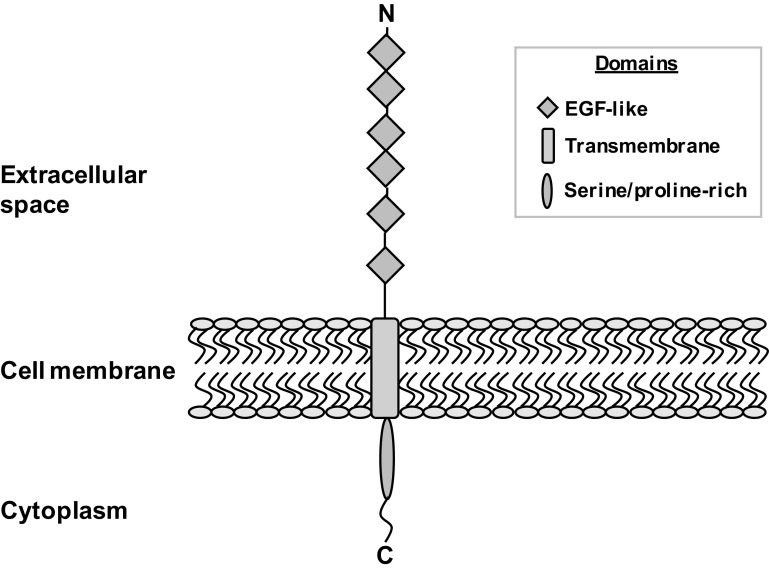
Diagrammatic representation of the structure of SCARF1. SCARF1 is a type I transmembrane protein consisting of three major domains: (1) an N-terminal extracellular region comprising of several epidermal growth factor (EGF)-like domains (blue diamonds); (2) a short transmembrane domain (pink rectangle) which spans the phospholipid bilayer (represented by the yellow ovals and ‘S’-shaped curves) of the host cell; and (3) a relatively long cytoplasmic and C-terminal tail region (red oval), which is rich in serine and proline residues. ‘N’ is representative of the amino (N)-terminus and ‘C’ represents the carboxyl (C)-terminus. (Color figure online)

Scavenger receptors are a large super family of proteins which are defined by their ability to bind and endocytose a vast range of ligands, eliciting their ‘scavenging’ of unsolicited endogenous and exogenous products [1]. Surprisingly, ligand affinity is often shared by a number of scavenger receptors, despite the different classes (A–J) showing little or no structural homology [1, 2]. Scavenger receptor class F, member 1 (SCARF1 or SR-F1 [3]), also known as scavenger receptor expressed by endothelial cells (SREC-I), is an 86 kDa type I transmembrane protein which contains several epidermal growth factor (EGF)-like domains in its extracellular region, a short transmembrane domain, and a long, serine- and proline-rich cytoplasmic tail [4] (Fig. 1). SCARF1’s long cytoplasmic tail is highly unusual for a scavenger receptor and the only other scavenger with such an exaggerated intracellular domain is the SCARF1 homologue, and second member of the class F family, SCARF2 [5]. The length of their cytoplasmic domains is highly suggestive of a role in intracellular signaling; however, this function is yet to be elucidated. SCARF1 is a highly evolutionarily conserved scavenger receptor, as its extracellular domain shows a significant sequence homology with the *Caenorhabditis elegans* scavenger receptor, CED-1 [6], a receptor which plays a key role in the homeostasis and innate immunity of *C. elegans* [6, 7]. Its homology with such a key protein in *C. elegans* is highly suggestive of an important role for SCARF1 in mammalian biology and, unlike the majority of scavenger receptor-deficient mouse lines which do not exhibit a resting state phenotype, the importance of SCARF1 becomes apparent in SCARF1-deficient (SCARF1^−/−^) mice which spontaneously develop systemic lupus erythematosus (SLE)-like autoimmune disease from 20 weeks of age. This autoimmune disease phenotype results from a significantly impaired clearance of apoptotic cells from key immunological organs, such as the spleen, and manifests in SLE-like symptoms, such as increased production of autoantibodies, splenomegaly and larger germinal centres with increased CD4^+^ T cells and B cells, severe dermatitis, and nephritis [8].

SCARF1 was first cloned from human umbilical vein endothelial cells (HUVEC) [4], but has since been shown to be expressed in a number of different cell types, including sinusoidal endothelial cells [9, 10], dendritic cells [8, 11], macrophages [8, 10], epithelial cells [12, 13], and B-1 cells [8]. However, the majority of studies exploring SCARF1 functionality have utilised transfected cell lines engineered to ectopically express the scavenger in vitro and very few have used primary cells which naturally express SCARF1 or in vivo approaches. In addition, the early studies on SCARF1 showed high transcriptional expression in a wide range of major human tissues, such as heart, kidney, liver, lung, and spleen [5], and this was later corroborated in murine tissues [8, 14]; however, there has been a distinct lack of studies exploring its expression and cellular distribution at the protein level in these tissues. Indeed, to date, there has only recently been one study which has thoroughly characterised the expression and cellular distribution of SCARF1 in both normal and chronically diseased human liver tissues [10]; therefore, there is currently a huge void in our knowledge of SCARF1 biology in human tissues and cells.

### Soluble SCARF1

Many scavenger receptors are known to exist in general circulation in a soluble form, which is often released via cell surface cleavage by exofacial proteases [1], and SCARF1 is no exception as it was recently shown for the first time that a truncated (~ 60 kDa) soluble form (sSCARF1) is the major species detectable in human serum [10]. Interestingly, the sSCARF1 was also detected in chronically diseased human liver tissues, but was absent from normal tissues; it was speculated that normal tissues did not contain the unknown protease(s) which were responsible for the cleavage of SCARF1 [10]. The functions of soluble scavenger receptors remain largely unknown, but their levels are often regulated in correlate with the extent of disease and so some, such as CD36 and CD163, exhibit the potential to become biomarkers [15, 16]; nevertheless, sSCARF1 did not seem to be regulated in chronic liver disease patients compared to normal controls [10]. Nevertheless, it would be interesting to explore serum levels of sSCARF1 in a range of patient cohorts with other inflammatory diseases known to involve scavenger receptors, such as atherosclerosis or Alzheimer’s disease, to further investigate its potential as a biomarker.

## Functions of SCARF1

As is a prerequisite of scavenger receptor classification, SCARF1 is a highly promiscuous receptor and has been shown to bind a wide range of endogenous and exogenous ligands (Table 1). The functions of SCARF1 in relation to these ligands are discussed below.

**Table 1:** Known SCARF1 ligands

Ligand	References
Endogenous	
SCARF1	[5]
SCARF2	[5]
Modified low-density lipoproteins (oxLDLs and AcLDLs)	[4]
Apoptotic cells (via complement factor C1q)	[8]
Heat shock proteins (Hsp70, Hsp90, Hsp110)	[11, 17–19]
Calreticulin	[20]
Tamm–Horsfall protein	[21]
Pancreatic zymogen granule protein 2 (GP2)	[22]
Protein phosphatase 1α CD4^+^ T cells (*unknown ligand*)	[23] [10]
Exogenous	
Bacterial antigens	
Teichoic acid (*S. aureus*)	[12]
Lipopolysaccharide (LPS)	[10, 24]
Outer membrane protein (Omp)A (*K. pneumoniae*)	[25]
Porin (Por)B_IA_ (*N. gonorrhoeae;* via Gp96)	[13]
Viral antigens	
Non-structural protein (NS)3 (*Hepatitis C virus; HCV*)	[26]
dsRNA	[27]
Fungal antigens	
β-Glucan (*C. albicans* and *C. neoformans*)	[6]

A number of endogenous and exogenous ligands have been described in the literature to date and are listed above

*S. aureus: Staphylococcus aureus, K. pneumonia: Klebsiella pneumonia, N. gonorrhoeae: Neisseria gonorrhoeae, C. albicans: Candida albicans, C. neoformans: Cryptococcus neoformans*

### Low-density lipoprotein (LDL) binding

The first scavenger receptor was described in the late 1970s by Brown and Goldstein, and was defined by its ability to bind and subsequently internalise low-density lipoproteins (LDLs) [28, 29]; therefore, upon identification of a potential new scavenger receptor, its ability to bind LDLs is often the first test to be undertaken. Consequently, in the original paper in which SCARF1 was cloned, they demonstrated its binding ability to modified LDLs, including acetylated (Ac-) and oxidized (ox)LDLs [4]. Subsequently, SCARF1 has also been shown to bind to carbamylated (c)LDLs [30]. As a result of their function as LDL receptors, a number of scavenger receptors, such as CD36 and LOX-1, are known to play an important role in the pathophysiology of atherosclerosis [31–33]; however, the contribution of SCARF1 to this inflammatory cardiovascular disease has yet to be explored in any capacity.

### Apoptotic cell clearance

Given that more than 10^9^ cells undergo apoptosis in the human body per day, a rapid and immunologically ‘clean’ removal of apoptotic cells by neighbouring phagocytic cells is essential for the maintenance of homeostasis and avoidance of inflammation [34]. The first indications of SCARF1’s involvement in this vital homeostatic process were the early findings of Berwin and colleagues who demonstrated its binding interaction with calreticulin [20], an important chaperone protein heavily implicated in the active uptake of apoptotic cells [35]. Following this, a seminal study by Ramirez-Ortiz et al. demonstrated that SCARF1^−/−^ mice spontaneously develop SLE-like autoimmune disease at 20 weeks of age as a consequence of impaired apoptotic cell clearance, thus highlighting the prominence of SCARF1 in this homeostatic process [8]. Their study elegantly demonstrated that SCARF1-expressing splenic dendritic cells, sinusoidal endothelial cells, and macrophages all significantly contributed to the clearance of apoptotic cells through binding to the complement factor, C1q [8], which, in turn, binds to the ‘eat-me’ signal, phosphatidylserine, presented on the surface of cells undergoing the early stages of apoptosis [36]. Nevertheless, this study was undertaken in the relatively artificial context of transgenic mice completely lacking SCARF1 expression in all tissues and so its translational relevance to the human disease still remains to be seen.

### Innate immunity

SCARF1 has been shown to bind a diverse range of bacterial (both Gram-positive and Gram-negative), viral, and fungal antigens (Table 1), thus implicating it in host innate immunity; however, whether its role is beneficial or detrimental is still relatively unclear. Two studies have implicated SCARF1 in adherence of bacteria on epithelial tissues and have suggested that the bacteria may utilise the scavenger receptor to promote their dissemination from the primary infection site to systemic circulation and distal tissues. One of these studies identified epithelial-expressed SCARF1 as a highly selective receptor for the *Neisseria gonorrhoeae* porin protein, PorBIA, in in vitro bacterial adhesion assays [13], whilst the other elegantly showed, both in vitro and in vivo, that *Staphylococcus aureus* is able to avidly bind SCARF1 expressed on nasal epithelial cells via its wall teichoic acid (WTA) [12]. However, although these studies showed that antibody blockade of SCARF1 on the epithelial layers effectively prevented adhesion of their respective bacterial strains, their conclusions that SCARF1 may promote bacterial invasion may be unfounded, as they both failed to consider the bacteria–SCARF1 interaction from a host perspective. Epithelial tissues are well characterised to evoke effective and efficient host immunological responses when under pathogenic attack [37, 38], yet neither study explored the SCARF1-mediated stimulation of the epithelial tissues, which could potentially facilitate these host responses.

It has also been suggested that SCARF1 could play an indirect role in the capture and uptake of bacteria via two homologous endogenous ligands, the Tamm–Horsfall protein (THP) [21] and pancreatic zymogen granule protein 2 (GP2) [22]. Although the physiological functions of THP and GP2 are yet to be elucidated, both have been shown to directly bind bacteria in vitro and could, therefore, act as opsonising or bridging agents which then bind SCARF1 on nearby immune cells and mediate the relevant immunological response; however, this is purely speculation on the author’s part and clearly further research is required.

In addition, a number of studies have shown evidence to suggest that SCARF1 acts in cooperation with a family of essential pattern recognition receptors (PRRs), called the Toll-like receptors (TLRs), to bind a range of pathogen-associated molecular patterns (PAMPs) and elicit a proinflammatory immunological response. A study by Jeannin *et al*. demonstrated that SCARF1 was able to directly bind an outer membrane protein (OmpA) isolated from *Klebsiella pneumonia*, but required the presence of TLR2 to elicit the release of the potent neutrophil chemoattractant and proinflammatory cytokine, IL-8, from transfected Chinese hamster ovarian (CHO) cells [25]. A seminal study by Means and colleagues also demonstrated in vitro and in vivo that SCARF1 is able to mediate binding and uptake of the fungal pathogens, *Cryptococcus neoformans* and *Candida albicans*, via β-glucan, but, again, the presence of TLR2 was required to elicit macrophage activation and the initiation of a proinflammatory immune response [6]. Furthermore, it has also been shown that SCARF1 again cooperates with TLR2 to induce myeloid cell activation in response to the hepatitis C virus (HCV) protease, non-structural protein (NS)3 [26]. More recently, Murshid and colleagues have also shown that the presence of SCARF1 can augment the TLR-mediated activation of the NF-κB pathway and ultimately the release of proinflammatory cytokines, such as IL-8, IL-6, and TNFα, in cells stimulated with either the TLR3 ligand, poly I:C (dsRNA) [27], or the TLR4 ligand, lipopolysaccharide (LPS) [24].

As mentioned previously, much of this work has utilised in vitro experimentation in cell lines ectopically expressing SCARF1; and, although these results are promising, their validation in primary cells which naturally express SCARF1, in more sophisticated multi-cellular in vitro models (e.g., organoids), or in murine models of infection is required to fully appreciate the role of SCARF1 in the innate immunity.

### Adaptive immunity

In addition to being implicated in the innate immunity, there is evidence to suggest that SCARF1 is also involved in the adaptive immunity and facilitates the uptake of chaperone-bound antigens and their subsequent antigen presentation to T cells [11, 17–19]. These studies have demonstrated that SCARF1 expressed on antigen presenting cells (APCs), such as dendritic cells, is able to internalise heat shock protein (Hsp)-bound antigens and load them, through the endosomal network, onto the cross presentation (MHC class I-mediated) [19] or class II presentation (MHC class II-mediated) [11, 17] pathway and subsequently active the adaptive immunity. Indeed, these studies went on to show that SCARF1-mediated presentation of Hsp-bound antigens on APCs was able to prime the activation of CD8^+^ or CD4^+^ T cells [11, 17, 19], dependent on the presentation pathway followed. However, it is currently unclear as to whether the choice in presentation pathway is purely stochastic in nature or if it is an active ‘choice’ by the APC mediated in some way by SCARF1 and future studies should consider this phenomenon.

### Leukocyte adhesion molecule

The leukocyte adhesion cascade is a multi-step process which facilitates the extravasation of leukocytes from circulation into inflamed tissues during injury or infection, with the primary aim of eliminating the inflammatory trigger and/or contributing to tissue repair. The adhesion cascade is mediated by a large number of chemoattractant cytokines (chemokines) [39] and adhesion molecules [40], and several endothelial-expressed scavenger receptors, such as stabilin-1 and lectin-like oxidized low-density lipoprotein receptor-1 (LOX-1), have previously been shown to directly bind leukocytes and facilitate their recruitment to various vascular endothelia [41–47]. An early study demonstrated that SCARF1-expressing cells can form moderate homophilic cell–cell interactions with each other and strong heterophilic interactions with SCARF2-expressing cells [5], an effect which implied the potential for SCARF1 to also mediate leukocyte recruitment. Recently, my lab characterised SCARF1 expression in primary human hepatic sinusoidal endothelial cells (HSEC) and, utilising a combination of recombinant proteins, HSEC and siRNA knockdowns in HSEC, we were able to robustly demonstrate that SCARF1 plays a role in the selective recruitment of CD4^+^ T cells to the sinusoidal endothelium under physiological shear stress conditions [10]. In this study, we proposed that SCARF1 acts in the firm adhesion step of the leukocyte adhesion cascade, showing SCARF1^+^ adhesive cups form on the surface of the HSEC [10]; however, we did not explore the possibility SCARF1’s involvement in the transendothelial migration step of the cascade and future investigations will explore this. We also ruled out the possibility that this SCARF1-lymphocyte binding is mediated through homophilic interactions with itself or through heterophilic interactions with its homologue, as CD4^+^ T cells do not express SCARF1 [10] or SCARF2 (unpublished data). Therefore, the lymphocyte-expressed ligand of SCARF1 is yet to be identified and screening experiments could be employed to determine this in future investigations. Furthermore, our demonstration of SCARF1’s adhesive function has only been in in vitro systems and so future studies will also aim to validate this in vivo, utilising a combination of liver injury models and intravital imaging in SCARF1^−/−^ mice.

## Conclusions

SCARF1 is known to bind a wide range of ligands both endogenous and exogenous in nature; consequently, a number of biological roles in homeostasis, immunity, and disease pathology have previously been suggested. However, the majority of studies undertaken thus far have relied heavily on in vitro experiments involving the ectopic expression of SCARF1 in cell lines and, whilst the validity and value of these studies is not being called into question here, this review highlights the distinct lack of research with regards to SCARF1 which utilises naturally expressing primary cells and ex vivo or in vivo approaches. Consequently, the cellular and tissue expression and function of this highly promiscuous scavenger receptor still remain a mystery and future studies should aim to reduce this significant deficit.
